# Study on the Factors Associated with Postpartum Visits in Rural China

**DOI:** 10.1371/journal.pone.0055955

**Published:** 2013-02-27

**Authors:** Hua You, Jianhua Chen, Lennart Bogg, Yuan Wu, Shengnan Duan, Chiyu Ye, Xiaofang Liu, Hai Yu, Vinod Diwan, Hengjin Dong

**Affiliations:** 1 Center for Health Policy Studies, School of Medicine, Zhejiang University, Hangzhou, China; 2 Institute of Global Health, Karolinska Institutet, Stockholm, Sweden; 3 School of Health, Care and Welfare, Malardalen University, Västerås, Sweden; 4 Institute of Public Health, Heidelberg University, Heidelberg, Germany; Universidade Federal do Acre (Federal University of Acre), Brazil

## Abstract

**Background:**

Postpartum visits (PPVs) have been advocated as a way to improve health outcomes for mothers and their infants, but the rate of PPVs is still low in rural China. This study aims to investigate the utilization of PPVs and to explore the factors associated with PPVs in rural China. Parity is the most concerned factor in this study.

**Methods:**

A cross-sectional household survey was performed in two counties of Zhejiang province. Questions include socio-economic, health services and women's delivery data. Chi-square tests and multivariate logistic regression analyses were performed to identify factors associated with PPVs.

**Results:**

223 women who had a delivery history in the recent five years were enrolled in analyses. 173 (78%) of them were primiparous. Among the primiparous women, 43 (25%) had not received any PPVs. The majority, 27 (55%) of the 49 multiparous women, had not received any PPVs. Multiparous women were less likely to receive PPVs than primiparous women. Among 223 puerperal women, 47 (21%) had been compensated for delivery fee expenses. Women who received compensation were found to be more likely to receive standard (at least 3) PPVs.

**Conclusions:**

It was found that women with “second babies” were less likely to use PPVs. This could be an unintended consequence of the “one-child policy”, due to fear that contact with public health facilities could result in sanctions. This phenomenon should be taken seriously by government in order to improve the health of babies and their mothers. Financial compensation for delivery fee charges can improve the use of PPVs, thus free-of-charge delivery should be promoted.

## Introduction

The postpartum period starts about an hour after the delivery of the placenta and lasts the following six weeks.[1] During this period postpartum visits (PPVs) have been advocated as a way to improve outcomes for mothers and their infants. It is believed to offer an important opportunity to assess the physical and psychosocial well-being of them [2]–[5]. However, postpartum care continues to be the “least emphasized” element in maternity services [6], [7], although it is vital for the reduction of diseases and deaths amongst mothers and neonates [8]. Studies in some developing countries have shown that low utilization of postnatal care [9]–[11]. China has made great achievements over the past two decades in reducing its maternal mortality rate and its under-five child mortality rate [12]. However, a study on the utilization of maternal health services revealed that the rate of postpartum visits is at a relatively low level also in China and especially so in the rural areas [13]. The Ministry of Health in China has set a target that at least 70% of urban women and 60% of rural women should receive three postpartum visits at home by health-care providers [14], whereas studies reveal that this is not happening in practice. The 3rd National Health Service Survey (NHSS) in 2003 showed that the rate of women having at least one PPV was 60% in urban areas, 52% in rural areas and only 37% in the poorest rural areas [15]. A recent survey in rural areas of Anhui Province found that the percentage of women having one or more postnatal visit was only about 4% [16].

Previous studies have identified a number of factors that explained the low rate of postnatal care from the service supply side. It is generally related to unavailability, inaccessibility and poor quality of health services [16], [17]. However, there is a lack of studies that have analyzed the low rate of PPVs from the demand side, what makes women demand postpartum care and what makes them less willing to receive postpartum visits [11]. Notwithstanding the Chinese MCH policies, the PPV practices vary in different groups of rural women. No published studies so far have analyzed how the policies influence the demand and utilization of PPV.

This paper aims to explore factors associated with postpartum visits services in rural China, focusing on the association with the parity under the conditions of the existing “one child policy” context.

## Methods

### Study sites

We carried out this study in Zhejiang province, which is located in the eastern coastal area of China with about 47 million population (2010). The socio-economic indicators are at the top level among all provinces in China. The surveys were performed at two counties in Ningbo, the second largest city in Zhejiang province. The two counties have average socio-economic indicators in Zhejiang province and are the community study sites of Zhejiang University.

County A is located on the northwest of Ningbo city, with 6 streets and 15 towns, an area of 1527 km^2^ and a population of about 850,000. County B is located on the south of Ningbo city, with 5 streets and 6 towns, an area of 1268 km^2^, a population of about 480,000.

### Sampling

The sampling methods were the same as NHSS in 2008 (Zhejiang province sample, Ningbo was selected as the sole sample city in Zhejiang province). A multi-stage stratified cluster random sampling technique was used to choose the sample in Ningbo. First, two counties (county A and B) in Ningbo were chosen randomly. Then, five towns were drawn from each county, and two villages were drawn from each town randomly again. Finally, the number of the households drawn from each village was determined according to the proportion of the number of households [18]. A total of 2000 households were enrolled in this survey.

### Data collection

Two specific project offices were set up at the two counties' Center for Disease Control and Prevention (CDC). The offices were in charge of the training for the survey instructors and investigators, organized the investigation and carried out the quality control under the supervision of Zhejiang University. The household survey was carried out during June to August in 2011. The investigators were local medical personnel trained by researchers from Zhejiang University.

After expressing the informed consent, all members of a household were interviewed at home one by one by the investigators. The questionnaires were checked and quality was controlled by survey supervisors who were professionals from the local county CDC. Ethical approval was obtained from the Medical Faculty Ethics Committee of Zhejiang University.

The questionnaire from NHSS in China (2008 version) was used. This paper only used the general household information and the information of women who had childbirth history during the recent five years. It consisted of the characteristics of those female respondents (age, education, employment, household income), the situation of last time delivery (delivery method, parity), the characteristics of the neonates (sex, birth weight, being premature labor or not), childbirth compensation (a subsidy for childbirth fee expenses for rural families) and postpartum visits.

Postpartum visit was the dependent variable. The respondents were asked, “During 6-week after delivery, how many times did you receive postpartum visits?”

### Statistical analysis

The data was analyzed by giving the following hypotheses:

1. receiving postpartum visits or not is influenced by the women's basic characteristics. It is assumed that low education, unemployed, low household income and multiparous birth can increase non-use of PPV; caesarean, premature labor, low birth weight, male baby and compensation for childbirth can increase the PPV use.
2. the number of postpartum visits is also influenced by women's socio-economic factors. It is assumed that high education, the employed, high household income, male baby, low birth weight, premature labor, caesarean and compensation for childbirth can increase standard use of PPVs.

In the data analysis, we first dichotomized the responses to non-use or use of postpartum visits among all respondents in order to find the risk factors associated with nonuse of PPV; then among those who had been visited, we dichotomized the responses to standard use of PPV or not (three or more visits were defined as standard use since minimum three visits are required by Chinese Government policy) in order to find what influences standard use of postpartum visits.

At first, the Chi-square tests were used in univariate analyses, then multivariate logistic analyses were used for the two dependant variables, respectively, nonuse or use of PPV and standard use of PPV or not. According to the theoretical hypotheses, the independent variables included the characteristics of the mothers, the situation of their last time delivery, the characteristics of the neonates, and compensation for childbirth fees. In the multivariate analyses, age was removed because it had a strong correlation with parity; the significance level in the analyses were set as 0.05 and enter method was selected. Double data entry was performed and data analysis was performed using SAS version 9.2 (SAS Institute Inc, Cary, NC, USA).

## Results

### General information of the sample

A total of 5513 subjects from 2000 households were interviewed, but 575 respondents were excluded because of their incomplete information. The response rate is 90% after check-control and re-investigation measures. There are no statistically significant differences in ratio of parity (χ^2^ = 0.78, *P* = 0.38) and in ratio of compensation for childbirth (χ^2^ = 0.01, *P* = 0.93) between incomplete and complete cases. It is also found that the trend of using PPV between incomplete and complete cases is the same, higher ratio of nonuse PPV in multiparous women than primiparous women (*P* = 0.24, Fisher's exact test). The excluded data do not cause bias.

The majority of respondents (98.8%) were Han (the majority ethnicity). Half (50.5%) were males. The median age was 49 years old, and median of annual household income was 40,000 Yuan (RMB).

223 women who had a live birth recently were included in this study. The average age was 29.3±4.8 years old. The annual household income varied from 10,000 to 500,000 Yuan (RMB) with a median of 50,000 Yuan. 173 (78%) of the puerperal women were primiparous. Among 223 women, 47 (21%) had been compensated for delivery fee expenses (Table 1).

**Table 1:** Characteristics of the study subjects and their postpartum visits.

Characteristics		Frequency (%)	PPV use				Standard PPV use			
			Yes (%)	No (%)	?^2^	*P*	Yes (%)	No (%)	?^2^	*P*
Education (n = 223)										
>Secondary level		96(43.0)	77(80.2)	19(19.8)			20(26.0)	57(74.0)		
≤Secondary level		127(57.0)	76(59.8)	51(40.2)	10.53	0.001	18(23.7)	58(76.3)	0.11	0.74
Employment (n = 223)										
Yes		195(87.4)	134(68.7)	61(31.3)			31(23.1)	103(76.9)		
No		28(12.6)	19(67.9)	9 (32.1)	0.01	0.93	7(36.8)	12(63.2)	1.68	0.20
Annual household income (n = 223)										
>50000 Yuan		110(49.3)	83(75.5)	27(24.5)			22(26.5)	61(73.5)		
≤50000 Yuan		113(50.7)	70(61.9)	43(38.1)	4.72	0.03	16(22.9)	54(77.1)	0.27	0.60
Delivery method (n = 217)										
Vaginal		114(52.5)	82(71.9)	32(28.1)			18(22.0)	64(78.0)		
Caesarean		103(47.5)	71(68.9)	32(31.1)	0.23	0.63	20(28.2)	51(71.8)	0.79	0.38
Parity (n = 222)										
Primiparous		173(77.9)	130(75.1)	43(24.9)			32(24.6)	98(75.4)		
Multiparous		49(22.1)	22(44.9)	27(55.1)	16.18	<0.001	6(27.3)	16(72.7)	0.07	0.79
Premature labor (n = 209)										
Yes		33(15.8)	26(78.8)	7(21.2)			5(19.2)	21(80.8)		
No		176(84.2)	120(68.2)	56(31.8)	1.49	0.22	29(24.2)	91(75.8)	0.29	0.59
Sex of neonates (n = 217)										
Male		113(52.1)	82(72.6)	31(27.4)			19(23.2)	63(76.8)		
Female		104(47.9)	71(68.3)	33(31.7)	0.48	0.49	19(26.8)	52(73.2)	0.26	0.61
Birth weight of neonates (n = 216)										
<2500 g	11(5.1)		8(72.7)	3(27.3)			4(50.0)	4(50.0)		
≥2500 g	205(94.9)		144(70.2)	61(29.8)	0.03	0.861	34(23.6)	110(76.4)	2.45	0.121
Compensation for delivery fee expenses (n = 222)										
No	175(78.8)		115(65.7)	60(34.3)			23(20.0)	92(80.0)		
Yes	47(21.2)		38(80.9)	9(19.1)	3.96	0.05	15(39.5)	23(60.5)	5.80	0.02

1 It was results of Likelihood ratio tests.

In this study, we focused on if women with the second baby had different use of PPVs from the women with the first one. There are no significant differences in most characteristics between primiparous women and multiparous women except age and education (Table 2).

**Table 2:** Characteristics of women with different parity.

Characteristics	Primiparous	Multiparous	?^2^	*P*
	Frequency(%)	Frequency(%)		
Age^1^	27.9±3.7	34.1±5.1	7.92	<0.001
Annual household income^2^	55000(40000–80000)	50000(30000–95000)	0.08	0.94
Education				
>Secondary level	84(48.6)	11(22.4)		
≤Secondary level	89(51.4)	38(77.6)	10.63	0.001
Employment				
Yes	150(86.7)	44(89.8)		
No	23(13.3)	5(10.2)	0.33	0.57
Delivery method				
Vaginal	87(51.8)	26(54.2)		
Caesarean	81(48.2)	22(45.8)	0.09	0.77
Premature labor				
Yes	25(15.5)	8(17.0)		
No	136(84.5)	39(83.0)	0.06	0.81
Sex of neonates				
Male	92(54.8)	21(43.8)		
Female	76(45.2)	27(56.2)	1.82	0.18
Birth weight of neonates				
<2500 g	8(4.8)	3(6.4)		
≥2500 g	160(95.2)	44(93.6)	0.20	0.66
Compensation for delivery fee expenses				
No	135(78.5)	39(79.6)		
Yes	37(21.5)	10(20.4)	0.03	0.87

1 the results for age were mean and standard deviation, a t-test was used to compare two means.

2 the results for household income were median and quartile range, a t-test was used to compare logarithms of two medians.

### Postpartum visits

Among 223 women, 31.4% of them never received any PPVs, 51.6% received one or two visits, and 17.0% got PPVs three or more times. Among the primiparous women, 43 (25%) had not received any PPVs. Among the multiparous ones, the majority, 27 (55%) had not received any PPVs (Table 3). The difference in PPVs between primiparous and multiparous women was statistically significant at *P*<0.001. Multiparous women have lower access to PPVs and less standard use of PPVs even obtaining the services.

**Table 3:** Difference of PPVs between primiparous and multiparous women.

Parity	Number of PPVs (times)			?^2^	*P*
	0	1∼2	3 or more		
	Frequency(%)	Frequency(%)	Frequency(%)		
**Primiparous**	43(24.9)	98(56.6)	32(18.5)		
**Multiparous**	27(55.1)	16(32.7)	6(12.2)	16.23	<0.001
**Total**	70(31.4)	114(51.6)	38(17.0)		

n = 222 after exclusion of missing data.

### Factors influencing non-use or use of postpartum visits

A major objective of the study was to find factors influencing nonuse or use of PPVs. In the univariate analysis, we found that multiparous parity was a risk factor for nonuse of PPV (χ^2^ = 16.18, *P*<0.001). The univariate analysis also showed that women with low education level (χ^2^ = 10.53, *P* = 0.001), or with low household income reduced PPV use (χ^2^ = 4.72, *P* = 0.03). Compensation for childbirth fee expenses more likely improved PPV (χ^2^ = 3.96, *P* = 0.05) (Table 1).

Except the aforementioned factors, we also found the other characteristics, which included unemployed, caesarean delivery, no premature labor, female neonates and normal birth-weight, were not significantly associated with non-use of PPV. (Table 1).

The multivariate analysis results showed, however, that only parity was significantly associated with non-use or use of PPVs. The multivariate-adjusted odds ratio for nonuse of PPV was 3.69 for women who were primiparous (Table 4). The Chi-square for Hosmer & Lemeshow test of this model was 6.38 (*P* = 0.5), and the fit of the multivariate logistic regression model was good. (The Hosmer-Lemeshow test is a statistical test for goodness of fit for the logistic regression model. A large value of χ^2^ with *P*<0.05 indicates poor fit and a small χ^2^ indicates a good logistic regression model fit [19]).

**Table 4:** Factors associated with nonuse and standard use of postpartum visits (multivariate analysis).

Characteristics	Nonuse of PPVs^1^		Standard use of PPVs^2^	
	OR (95%CI)	*P*	OR (95%CI)	*P*
Education				
>Secondary level	1		1	
≤Secondary level	1.81(0.89–3.70)	0.10	1.56(0.64–3.82)	0.33
Employment				
Yes	1		1	
No	1.32(0.50–3.46)	0.57	1.57(0.49–5.02)	0.45
Annual household income				
>50000 Yuan	1		1	
≤50000 Yuan	1.50(0.75–2.98)	0.25	0.72(0.30–1.74)	0.47
Delivery method				
Vaginal	1		1	
Caesarean	1.31(0.69–2.50)	0.41	1.50(0.65–3.45)	0.34
Parity				
Primiparous	1		1	
Multiparous	3.69(1.78–7.65)	<0.001	1.14(0.36–3.66)	0.82
Premature labor				
Yes	1		1	
No	2.04(0.76–5.49)	0.16	1.84(0.52–6.60)	0.35
Sex of neonates				
Male	1		1	
Female	1.17(0.62–2.22)	0.62	1.23(0.53–2.86)	0.63
Birth weight of neonates				
<2500 g	1		1	
≥2500 g	1.04(0.23–4.67)	0.96	0.22(0.04–1.16)	0.07
Compensation for delivery fee expenses				
No	1		1	
Yes	0.63(0.25–1.57)	0.32	3.15(1.23–8.09)	0.02

1 n = 207 after exclusion of missing data for all covariates in multivariate analysis.

2 n = 144 after exclusion of missing data for all covariates in multivariate analysis.

### Factors influencing standard use of postpartum visits

The second objective of our study was to identify if the factors influencing the standard use of PPVs. We found that among all independent variables, only the variable “compensation for delivery fee expenses” had significant positive association with standard use of postpartum visits. The OR value was 3.15 in the multivariate analysis (Table 4). Women who received the compensation for childbirth fee expenses were more likely to receive standard postpartum visits than those who had not received subsidies (39.5% vs. 20.0%) (Table 1). Other variables such as education, household income, parity, the status of neonates were not found to be significantly associated with standard use of PPVs. The Chi-square for Hosmer & Lemeshow test of this model was 3.34 (*P* = 0.91), meaning the model's fitting well [19].

## Discussion

We found that about 70% of the women in the study population were visited by medical professionals for a postnatal check-up during the first 6-week following delivery. This rate is comparable with other estimates in rural China [13].

It is very important to note in our study that being multiparous links to non-use of postpartum visit. Being multiparous i.e. having more than one child, implies that they are not “only-child” families, as prescribed by the family planning regulation in China. This touches a particular and complicated issue in China because of the strict “one-child policy” in place since the late 1970s [20]–[21]. Most multiparous births are in breach of this policy. In order to control the total population number, China continues to advocate only one child for one couple. Pregnancies in violation of the policy sometimes result in recommendations to abort the fetus [22]–[24], or being result in the family being levied a “social rearing fee” as an economic penalty [25].

Yet, there are numerous observations of pregnancies in conflict with the family planning policies, especially in rural areas [20]. The latest national census in 2010 revealed the number of population without “hukou” (a household registration under the supervision of the Ministry of Public Security of P. R. China) was 13 million, equivalent to 1% of the total population, most of whom were born outside the “one child policy” [26]. The number of unregistered children in China could be higher since families with unregistered children are likely to avoid officials, including census enumerators.

Many “second baby” mothers are afraid to visit public hospitals since they fear sanctions. Covert deliveries lead to the families missing opportunities to receive normal and vital maternal services. Postpartum visits are among the neglected priority services. Utilization of postpartum care services is very low among multiparous women. Therefore, we suggest that special attention needs to be given to the situation of multiparous women to address the lack of access to maternal services also among the “one child policy” violators.

In addition, postpartum care receives more attention to at the first birth than at the subsequent births [27]. Some multiparous mothers may believe they have enough experiences of delivery and “doing the month” (a Chinese traditional calling for the convalescence during the postnatal period), and skills for breast-feeding drawn from the first birth. When giving birth to the next child, the postpartum visits are not taken as seriously as for the first delivery. This may be deduced as another reason leading to non-use of postpartum visits among the multiparous women.

Besides the parity, in the univariate analysis, we also found that lower education, lower household income and no compensation for delivery can significantly reduce the use of PPV; however, in the multivariate analysis, their effects disappeared. We further found that there are no significant differences in household income and compensation for delivery between primiparous and multiparous women, but there is significant difference in the education. It implies that household income, compensation for delivery and education have no direct effects on PPV use; however, education has indirect effect on the use through its direct positive effect on multiparous puerpera.

Even among those who receive PPVs, there are still majority receiving visits less than three times. Health visitors' multiple-postpartum-home-visits to low risk mothers can increase maternal physical and psychological health and may decrease use of emergency medical services for infants [28]. Of all the respondents, less than 20% received standard PPVs. It is much less than the recommendation (60%) from China's Ministry of Health [14].

For the factor significantly positively associated with standard use of PPV, compensation for delivery fee expenses is the sole one in our study. It increased the standard use although only a small fraction of the mothers obtained compensation. This compensation is a fixed subsidy exclusively for the delivery fee expenses and for rural women who participate in the New Rural Cooperative Medical Scheme (NCMS). Although the compensation is for hospital delivery fees specially, it is noteworthy that it also can influence demand and utilization of the postpartum services. A qualitative study in 2009 reported that some rural women acknowledged they would increase maternal health services utilization if they received some compensation [29]. Thus, compensation for or free delivery becomes very important because it can improve standard use of PPVs and protect women's health.

In summary, our results illustrate that rate of non-use of PPV is different between “the second babies” and “the first babies”, so it should be paid more attention to increase the PPVs among “the second babies”. We also find that financial compensation can help to improve the rate of utilization of standard postpartum visits. From the demand-side perspective, measures to ease the financial burden can really play a role to enhance maternal service utilization.

There are several limitations to this study. Firstly, this study used a sample drawn from the most developed province in China. The results will probably overestimate the national level of PPVs utilization. The conclusions can not represent the situation of the whole country because of the large socio-economic differences between regions and because of the decentralized nature of the health system in China. The comparative study between different regions should be encouraged in the future. Secondly, it was a cross-sectional study. We found some associations, but further studies are needed to confirm them. Thirdly, our study considers the demand-side perspective, while clearly supply-side factors also influence PPVs accessibility and utilization. Future research should examine the combined effects of demand-side and supply-side factors.
